# Neutrophil Elastase Activity Imaging: Recent Approaches in the Design and Applications of Activity-Based Probes and Substrate-Based Probes

**DOI:** 10.1155/2019/7417192

**Published:** 2019-06-12

**Authors:** Natacha Jugniot, Pierre Voisin, Abderrazzak Bentaher, Philippe Mellet

**Affiliations:** ^1^Centre de Résonance Magnétique des Systèmes Biologiques, UMR5536, CNRS, Université de Bordeaux, 33076 Bordeaux, France; ^2^INSERM, 33076 Bordeaux Cedex, France; ^3^Equipe “Inflammation et Immunité de l'Epithélium Respiratoire”—EA7426 Faculté de Médecine Lyon Sud, 69495 Pierre Bénite, France

## Abstract

The last few decades of protease research has confirmed that a number of important biological processes are strictly dependent on proteolysis. Neutrophil elastase (NE) is a critical protease in immune response and host defense mechanisms in both physiological and disease-associated conditions. Particularly, NE has been identified as a promising biomarker for early diagnosis of lung inflammation. Recent studies have shown an increasing interest in developing methods for NE activity imaging both in vitro and in vivo. Unlike anatomical imaging modalities, functional molecular imaging, including enzymatic activities, enables disease detection at a very early stage and thus constitutes a much more accurate approach. When combined with advanced imaging technologies, opportunities arise for measuring imbalanced proteolytic activities with unprecedented details. Such technologies consist in building the highest resolved and sensitive instruments as well as the most specific probes based either on peptide substrates or on covalent inhibitors. This review outlines strengths and weaknesses of these technologies and discuss their applications to investigate NE activity as biomarker of pulmonary inflammatory diseases by imaging.

## 1. Introduction

Degradome analysis indicates that protease and protease inhibitor genes represent more than 2% of total genes in human genome [1]. Proteases regulate a variety of physiological processes critical for life [2] including regulation of maturation, localization, activity and recycling of many proteins, modulation of protein-protein interactions, processing in signalization, transduction, and amplification of molecular signals. Proteases influence DNA replication and transcription, cell proliferation, migration, and differentiation, tissue remodeling, angiogenesis, wound repair, blood coagulation, digestion, ovulation, inflammation, necrosis, and apoptosis and immune response as well as pathogen clearance [3]. Accordingly, proteases are a major focus of attention for the pharmaceutical industry as potential drug targets or as diagnostic and prognostic biomarkers [4]. Since the function of proteases is to cleave proteins and peptides in response to biological, chemical, or physical stimuli, their action must be carefully orchestrated and strictly controlled for homeostasis purpose. Therefore, their actions are regulated at multiple levels [5]. Proteases are traditionally synthesized as inactive enzymes called zymogens that require activation process. They consist in subunit multimerization. For instance, dimerization of the Human Immunodeficiency Virus type 1 (HIV-1) protease subunits is an essential process for the acquisition of proteolytic activity, which plays a critical role in the maturation and replication of the virus [6]. Another way of regulation consists in proteolytic cleavage of precursors, as pancreatic serine proteases, activated for food digestion only when they reach the duodenum [7]. Others mechanisms consist either in blocking active proteases by endogenous inhibitors or in trapping them in a dedicated compartment such as mitochondria, specific apical membranes, and lysosomes, in which acidic pH participates also to enzyme activity containment. Particularly, mature protease neutrophil elastase (NE) is stored in specific neutrophilic granules in an active form that should be inactivated by inhibitors upon its extracellular release in the setting of inflammation or infection [8]. Therefore, if not correctly regulated, NE activity has the ability to damage host tissues leading to the development of pathologies as pointed below. Accordingly, noninvasive methods allowing direct and reliable monitoring of protease activity in the context of complex biological samples or in vivo especially in diseased situations are greatly needed for a diagnostic purpose. In this context, this review will focus on recent advances in NE activity detection, quantification, and applications both in vitro and in vivo.

### 1.1. Neutrophil Elastase: A Double-Edged Sword

NE (EC 3.4.21.37) is a 29 kDa serine protease of chymotrypsin family stored in azurophilic granules of polymorphonuclear neutrophils [9, 10] and released during neutrophil degranulation [11]. NE plays a relatively important role in neutrophil-mediated bacterial killing, and a cidal mechanism of the enzyme against Gram-negative bacteria has been elucidated [12, 13]. Furthermore, not only NE kills directly invading pathogens, but the enzyme fine tunes host inflammatory response for better pathogen eradication and its associated inflammation [14]. Normal inflammatory response triggers recruitment and activation of neutrophils toward the inflammation site. Neutrophil extracellular degranulation occurs and an overwhelming concentration of NE molecules over their endogenous inhibitors takes place. Protease activity is yet restricted in space and time by inhibitors in the tissue. Paradoxically, persistent or excessive inflammation as a result of prolonged exposure to stimulus has been found to be detrimental. NE with exacerbated elastolytic activity was shown to be one of the main lung destructive actors. Indeed, its large repertoire of substrates, particularly, elastin favorizes potent proteolysis [15]. NE activity can result in extensive lung tissue damages potentially leading to organ failure and death making inflammatory diseases a major health concern worldwide as well as an important economic burden [16]. Clinical studies have evidenced how an elevated concentration of NE correlates to acute lung injury [17], development of cystic fibrosis (CF) symptoms [18] as well as Chronic obstructive pulmonary disorders (stable and/or exacerbated COPD) [19], and bronchiectasis [20]. Moreover, it has been postulated that NE can contribute to the progress of lung cancer [21]. This correlation between NE activity level and disease progress has prompted researchers to investigate the use of NE as biomarker to diagnose and monitor pathological inflammations.

### 1.2. Pivotal Role of NE/Anti-NE Balance Status Imaging

A standard approach for looking at proteases in inflamed situations is the analysis of their transcript levels. Because of the posttranslational modifications, monitoring protease activity directly is more reliable to translate its roles in biological events. Another commonly used method for the quantification of NE is the immunodiagnostic from biological fluid samples. Antibody-related techniques yield information on total protease amount but lack the ability to differentiate between active and inactive enzyme forms. Methods to overcome such limitations tried to combine classical ELISA with the application of active-site inhibitors. A colorimetric active site-specific immunoassay (CASSIA) was described [22] for several serine proteases with arginyl specificity. Preferred protocols consist in using avidin capture of a biotinylated peptidyl arginyl chloromethyl ketone (CMK) and a specific antiprotease antibody recognition revealed by an enzymatic amplification step. In the case of a chymotrypsin-like specificity, a peptidyl inhibitor with a hydrophobic P1 amino acid side-chain can be used. Based on the CASSIA technique, cathepsin G activity was successfully detected [23]. It is thus largely conceivable that analog probes can be developed for a neutrophil-like activity detection. Nevertheless, a difficulty lies in the inescapable capture competition between protease-coupled probes and the uncoupled probes. Thus, to make sure that the enzyme activity measurement is not underestimated, a more complex assay is needed. It can consist in a preliminary assay in which uncoupled probe would be removed or in an increase of coated avidin concentration. Another method, termed ABRA-ELISA (Activity-Based probe RAtiometric-Enzyme Linked ImmunoSorbent Assay), was found particularly relevant on serine protease kallikreins for ovarian cancer diagnosis and could be applied to NE [24]. This strategy consists in combining the high throughput and high sensitivity of an ELISA-based detection with the advantage of an active-site inhibitor labeling. The proportion of the active form relative to the total concentration of the enzymatic biomarker (regular ELISA) would then be easily monitored in samples and may therefore serves as a novel diagnostic tool to quantify the active form in pathophysiological settings. However, these assays have limitations as the requirement for enzyme immobilization on a solid surface can result in its improper structural orientation or decreased reactivity with the target. It also needs collection of biological samples, requiring therefore an invasive intervention. Thus, methods for in vivo monitoring and imaging of NE activity in real time are studied.

Clinical symptoms are often undetectable at early stage of a disease, making diagnosis approach even more challenging. Nowadays, no reliable clinical methods exist to image deleterious NE proteolytic activity, which is again convincingly documented as a culprit in tissue destructive diseases. To address this concern, the field of functional molecular imaging represents an attractive and relevant tool [25]. The information quality provided is highly dependent on (*a*) the choice of the biomarker, (*b*) the physical, biochemical and pharmacological characteristics of the probe, and (*c*) the imaging modality [26] to characterize NE imbalance activity, especially during disease initiation phase.

**Figure 1:**
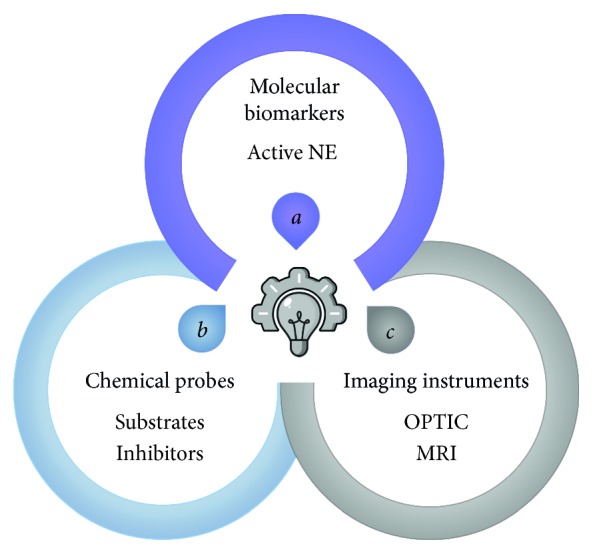
Key elements necessary for neutrophil elastase (NE) proteolytic imaging. The concept is to integrate molecular biomarker, chemical probes with imaging instruments to visualize, localize, and quantify NE activity for diagnosis (disease initiation and/or progression) and therapy follow-up of inflammatory processes. OPTIC: optical imaging; MRI: magnetic resonance imaging.

This review will discuss the design of novel probes and how their application in NE proteolytic activity imaging could become a reliable clinical diagnostic tool. On one hand, imaging agents binding their biological target with high specificity and affinity are worked out, including the design of substrate-based probes and activity-based probes. On the other hand, imaging instruments including optical imaging and Magnetic Resonance Imaging (MRI) methods should be able to detect disorders with high sensitivity and high resolution (Figure 1).

## 2. Molecular Technologies of NE Activity Imaging

The development of functional imaging technologies has led to the production of a myriad of molecular imaging agents for a variety of proteases [27–32].  Table 1 lists a series of probes specifically targeting NE activity and discussed in this review. Enzymatic constants, types of probe, and detection modality are indicated for each.

### 2.1. Activity-Based Probes

**Figure 2:**
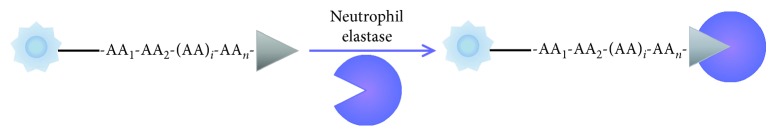
Overall principle of activity-based probe (ABP). Warhead (grey triangle) structurally matches with the target protease (purple). Active ABP can be detected by the tag (blue star). AA_1_-AA_*n*_ indicates amino acid position in the specific peptide.

Activity-based probes (ABPs) are low-molecular-weight molecule reporters designed to covalently bind a target enzyme as an active site reacting inhibitor and allow to visualize and localize active protease using fluorescence-based imaging modalities. All ABPs share a similar basic design, which incorporates elements required for targeting, modification, and detection of target proteins (Figure 2). Features that characterize ABP structures include (i) a reactive functional group termed as « warhead » that binds the catalytic residue of the enzyme active site, thus leading to the formation of covalent complex; (ii) a linker chain that has the basic function to separate the reactive functional group from the tag (for NE targeting, the linker corresponds to specific peptides matching its substrate binding pocket); and (iii) a reporter tag, often a biotin or fluorophore for the optical detection of the enzyme-probe complex. It should be noted that some isotope-based techniques were studied. For example, a radiolabeled aptamer-based inhibitor of NE coupled to ^99m^Tc has been used to image inflammation in a rat reverse passive Arthus reaction model [33]. In the same way, a human NE inhibitor (EPI-HNE-2) radiolabeled with ^99m^Tc has been used to visualize inflammation and infection in monkeys [34]. However, while nuclear imaging can be very sensitive, ionizing radiations limit its use in routine disease follow-up. Then, optical detection constitutes a judicious alternative.

ABPs appear very interesting for living organisms imaging applications. Indeed, one of the advantages of ABPs relies in the fact that the selectivity can be controlled both by the warhead and linker sequence. In this aim, focus on warhead and linker is more suitable over reporter tag.

#### 2.1.1. Warhead

Numerous peptide-based as well as nonpeptidyl inhibitors have been studied for NE specific studies [48]. Peptide CMK, like the most effective one MeO-Suc-AAPV-CMK, has proved to be very effective inhibitors of NE and particularly useful for structural studies [49, 50]. However, oligopeptides CMK are highly reactive and potentially toxic molecules, thus will never find a use in clinics [51]. They rather serve as a standard of comparison for developed inhibitors. ABPs have been proposed with a large repertoire of warhead designed to covalently link amino acid residue Ser195 of NE. Several groups reported interesting data about the influence of well-known classes of inhibitors used as warhead to study the biological functions of neutrophil serine proteases [52, 53]. Recently, Schulz-Fincke and colleagues highlighted the strong labeling capability of a sulfonyloxyphthalimide moiety as a new type of warhead that is linker-connected to a coumarin fluorophore [54]. The probe showed adequate fluorescence properties and suitable detection of NE in the presence of a large excess of cell lysate proteins with no detectable nonspecific interaction. The use of this probe against endogenous elastase from healthy donors-derived blood show promising results for further in vivo experiments.

#### 2.1.2. Linker

In addition to warhead development, recognition sequence of the linker adds to the probe specificity. This link controls the specificity of the inhibitor toward its target by a recognition element such as a peptidic sequence. The exploration of protease substrate specificity is generally restricted to naturally occurring amino acids, obviously limiting the degree of conformational space that can be surveyed. In their studies, Kasperkiewicz and coworkers reported the design of a hybrid natural and nonnatural peptidic substrate of NE, PK101, using a hybrid combinatorial substrate library profiling [35, 36]. That optimal substrate sequence exhibits astounding *k*_cat_/*K*_M_ surpassing the commonly used peptide sequence AAPV by more than 7000-fold and showed high selectivity (900-fold) to NE over the closely related protease Proteinase 3 (PR3). The substrate was converted in an extremely sensitive ABP and was applied to reveal NE activity in vitro during the process of neutrophil extracellular trap (NET) formation [35].

Although the development of selective ABP probes remains a challenge, we believe that incorporation of novel warheads mixed with original substrate design will enable a very specific targeting of biomarkers as NE. Nevertheless, by nature, ABPs bind only a single protease and generate only one detectable molecule per binding event. The detected signal is then directly proportional to the overall concentration of active protease, and detection of low-abundance proteins may be challenging.

### 2.2. Substrate-Based Probes

Signal amplification by multiple processing events can be successfully achieved by substrate-based probes. Indeed, one of the major benefits of using the turnover of substrate as a reporter is that a single active protease can process many substrates continuously leading thereby to signal amplification [55]. Bellow, we emphasize on the use of attractive substrate-based probes to NE activity monitoring.

#### 2.2.1. Substrate-Based Probes for Optical Imaging

**Figure 3:**
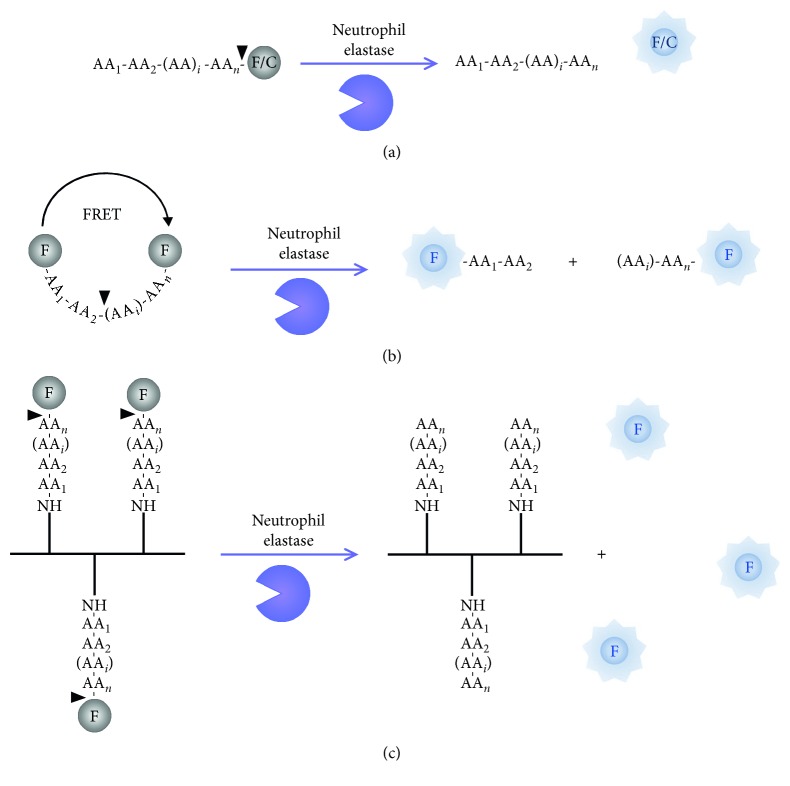
Protease-sensitive probes for optical imaging. (a) Fluorogenic (F)/chromogenic (C) enzyme-sensitive probe. One fluorescent or chromogenic molecule is bound to a peptide. Spectroscopic properties will be altered upon proteolysis. (b) Fluorophore-quencher type probe. Förster resonance energy transfer- (FRET-) based probes require a donor and an acceptor fluorophore pair each saturated on one side of the enzyme cleavage site. (c) Polymeric-peptide conjugate probe. Overabundance of fluorophores coupled to a polymer backbone via a peptide substrate. Black arrowheads depict cleavage site within the amino acid sequence.

Molecular imaging requires high-resolution and highly sensitive instruments to detect imaging agents that connect the imaging signal with the molecular event. Molecular imaging is easily performed using fluorescent probes enabling 3D images on small animals. A variety of organic fluorophores with emission wavelengths ranging from visible to near infrared region have been synthetized. These molecules can be modified with additional groups to optimize their inherent properties such as photophysical characteristics, solubility, cell permeability, toxicity, or enzyme specificity. Currently, three major types of activated fluorescent probes are used to monitor NE activity (Figure 3). The general approach is to design a substrate so that the prequench signal of the fluorophore can be turned « on » by protease activity.

*(1) FL/UV Enzyme-Sensitive Probe*. The fluorogenic/chromogenic probe design consists of a peptide attached at the C-terminus to a revelator, such as Amino-Methyl Coumarin (AMC, in vitro and in vivo fluorophore) or p-Nitro-Anilide (pNA, in vitro chromophore) (Figure 3(a)). Those probes are limited to a strong P1 interaction with two or three additional sites according to Schechter & Berger nomenclature [56]. Bieth and coworkers thoroughly studied the action of NE on substrates coupled to those two molecules varying in their peptidic chain length [57, 58]. Few years later, the group of powers developed a sensitive assay for NE activity inhibition involving the exploitation of the peptide sequence MeO-Suc-Ala-Ala-Pro-Val-CH_2_Cl, which has been reported as the most effective CMK inhibitor of NE [49]. Taking advantage of this sequence, they attached an AMC molecule to the C-terminal carboxyl group of the peptide sequence [59] (MeO-Suc-Ala-Ala-Pro-Val-AMC). The same year, MeOSuc-Ala-Ala-Pro-Val-pNA substrates were synthetized and analytically used as chromogenic substrate [37]. Currently, several synthetic NE substrates are commercially available. However, for routine assays, NE activity is measured using either fluorogenic peptide substrates such as *N*-methoxy-succinyl-Ala-Ala-Pro-Val-AMC or chromogenic peptide substrates such as *N*-methoxy-succinyl-Ala-Ala-Pro-Val-pNA. These are widely used to quantify NE and as a marker in inflammatory lung diseases, such as CF [60, 61].

*(2) Fluorophore-Quencher Type Probe*. Initially developed for caspase activity detection, many strategies use quenched fluorogenic substrates in accordance with the principle of Förster resonance energy transfer (FRET) [62]. FRET-based probe requires a pair of fluorophores, individually flanked at one each side of a peptide taking advantage of both the *P* and *P*′ specificity. In order for energy transfer to occur, the emission spectrum of the donor has to overlap with the excitation spectrum of the acceptor and both have to be located within a short distance from each other (<*R*_0,_ i.e., Förster distance at which the energy transfer efficiency is 50%). Cleavage of the linker drives away the two fluorochromes suppressing the energy transfer and resulting in an increase in the emission intensity of the donor and reducing or eliminating the acceptor emission (Figure 3(b)).

Potential utilization of FRET-based probes to monitor protease activity has been investigated in 2004 by Felber and colleagues [63]. They exposed an FRET system consisting of Cyan and Yellow fluorescent proteins (CFP and YFP, respectively) linked by a peptide. CFP-linker-YFP system was used for a variety of proteases. Based on Felber biosensor, Schulenburg et al. published a powerful FRET-based probe in 2016 [64]. They applied the CFP-linker-YFP system for NE with a linker containing a NE-recognition sequence. This probe exhibits about 200-fold more affinity than the chromogenic substrate reference (MeO-Succinyl-AAPV-pNA).

Other strategy consists in quenched FRET probes with improved specificity toward human and mouse neutrophil elastase, with the substrate sequence PMAVVQSVP [38]. The linker sequence in the construct is designed to be preferentially cleaved by NE while remaining resistant to other proteases including mouse PR3 [39]. The company PerkinElmer took advantage of this sequence to provide Neutrophil Elastase 680 FAST™ as a preclinical fluorescent activated sensor. It consists of the dedicated peptide sequence with two VivoTag-S680 fluorochromes, which are self-quenched and become highly fluorescent after cleavage by elastase. In 2011, Kossodo et al. used this substrate to image and quantify for the first time NE activity in mouse models with acute lung injury and response to treatments [65]. Neutrophil Elastase 680 FAST™ is now used widely as NE substrate and largely reported in the literature not only in lung inflammation but also in cancers [41, 66], arthritis [42, 43], and atherosclerosis [44].

So far, studies of NE activity have focused on free NE form. Interestingly, several reports demonstrated [9, 45, 67–69] and measured [67] the presence of a significant proportion of NE binding the anionic external plasma membrane of cells in an active form. Membrane-associated NE appears to be important in lung pathogenesis [70] and measuring its activity in vivo may be of great help in testing the activity of exogenous inhibitors since it was shown to be largely resistant to inhibition by endogenous inhibitors including as *α*1-antitrypsin and SLPI [45]. Particularly, the activity of membrane-associated NE pool may be relevant in early CF in young children for which no free NE activity is detectable in Bronchoalveolar Lavages (BALs) fluid despite structural evidence of lung disease by chest CT [71, 72]. Using the substrate sequence previously described, PMAVVQSVP [38], Schultz and coworkers generated FRET reporters for free NE (NEmo-1) as well as the lapidated form for plasma membrane insertion (NEmo-2) where two negative charges were introduced to prevent internalization into the cell [73]. For the first time, the role of membrane-associated NE activity was shown to correlate, ex vivo, with severity of lung disease in patients with CF and potentially other chronic neutrophilic lung diseases [18]. Interestingly, their results suggest that, in the microenvironment of CF airways, neutrophils acquire this activated configuration with increased membrane-associated NE activity even at low levels of inflammation, when free NE activity is still contained by endogenous antiproteases. Longitudinal studies in larger patient cohorts will be required to determine the predictive value of membrane-associated NE activity as a biomarker of disease severity and progression.

*(3) Polymeric-Peptide Conjugate Probe*. Alternatively, several studies used polymers modified with small organic fluorophores for proteolytic activity. Probes are developed utilizing a synthetic (i.e., dendrimer or polylysine) or protein (collagen, gelatin) [40] backbone to which a large number of reporters are attached via peptide linkers in close proximity to each other. Overabundance of fluorochromes anchored onto a polymer template through cleavable peptide substrate sequences in close proximity causes self-quenching. Upon peptide hydrolysis by proteases, fluorescence is restored and can be measured (Figure 1(c)).

A widely used series of probes is based on poly-l-lysine as a scaffold for conjugation of near-infrared fluorophores. Originally, Weissleder and colleagues first developed in 1999, a copolymer of poly-l-lysine and methoxypolyethylene glycol succinate (mPEGs) conjugated with cyanine dye (Cy5.5) for in vivo imaging [74]. Once internalized into cancer cells, 95% of the quenched fluorescence was recovered, resulting in a 12-fold increase in the fluorescent signal. Introduction of a selective peptide substrates between the polylysine backbone and the fluorophore allowed imaging of specific protease activities. By simply by replacing the peptides, imaging probes were then developed for detecting other disease-associated proteases such as cathepsins [75], MMPs [76, 77], caspases [78, 79], thrombin [80], or urokinase-type plasminogen activator [81, 82].

In 2005, Edwards and coworkers outlined an approach involving the use of cotton cellulose nanocrystal (CNC) fluorescent peptide conjugates as a support for a sensitive biosensor for NE and porcine pancreatic elastase (CNC-(O-C(O)Gly-NHC(O))succinyl-Ala-Pro-Ala-AMC) [83]. Relative to that shown by the tripeptide, peptide-CNC displayed 5-fold higher efficiency for NE as judged by a *k*_cat_/*K*_M_ value of 33 500 M^−1^·s^−1^.

There are however several drawbacks of using optical imaging: (a) substrate fluorescence quenching is not complete hence requiring long waiting times to eliminate nonspecific “blinding” light, (b) light tissue penetration is limited and prevents imaging of deeply seated tissues or skull, and (c) three-dimensional images are obtained by reconstruction.

#### 2.2.2. Substrate-Based Probes for Magnetic Resonance Imaging (MRI)

MRI appears particularly well suited to deliver exquisite anatomical details. It has a superior true 3D coding along with exceptionally good soft tissue contrast compared to optical imaging. MRI offers high spatial resolution and an unlimited depth penetration. Completely noninvasive, it allows simultaneous acquisition of anatomical structure and physiological function, particularly relevant for longitudinal follow-up involving multiple acquisitions. Nevertheless, the use of MRI is hampered by the limited sensitivity so far prevented clinical molecular imaging such as enzyme activity imaging. Thus, it requires the design of smart contrast agents and development of powerful signal amplification strategies.

*(1) Overhauser-Enhanced Magnetic Resonance Imaging (OMRI)*. To overcome this limitation, a particular imaging method based on Overhauser effect was developed to enhance NMR sensitivity by increasing the signal/noise ratio [46]. Briefly, OMRI is a double-resonance experiment transferring a part of the higher spin polarization of an unpaired electron to the environing water protons (through electron-proton Overhauser effect) which enhances the MRI signal that appears brighter on the final image. This method, called Overhauser-Enhanced Magnetic Resonance Imaging (OMRI), was improved to localize and image molecular processes. NMR signal enhancement was observed in mouse glioma thanks to intravenous injection of an original nonspecific spin probe design [84]. Probes with unpaired electron such as nitroxide molecules are stable enough in physiological conditions to be detected by OMRI, and they can be turned into enzyme activity probes. In 2014, for the first time, 3D visualization of proteolytic activities happened in vivo in mice using an on/off nitroxide-labeled with a 30 kDa elastin substrate probe [85]. High Overhauser enhancements of 10-fold were observed in the intestinal tract of mice after elastolytic activity on the probe.

**Figure 4:**
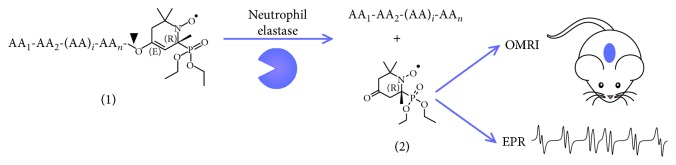
Proteolysis imaging by MRI. Proteolysis of peptide-locked nitroxide (1) into a free nitroxide (2) by neutrophil elastase creating high contrast in vivo by OMRI and EPR shift in vitro. Black arrowhead designates cleavage site in the amino acid specific sequence.

After such proof-of-concept, a *ß*-phosphorylated nitroxide-based probe was developed and turned into NE specific probe by adding a peptide moiety, MeO-Suc-AAPV, recognized by the target enzyme. The probe presented a *K*_M_ of 15 *μ*M and a *k*_cat_/*K*_M_ value of 930 000 M^−1^·s^−1^ [86]. It was designed such that its spectroscopic properties change upon removal of the peptide moiety by NE. This particular 6-peaks nitroxide has its resonant frequency shifted upon NE action (i.e, a change in its hyperfine coupling constants). It was tested in vitro by Electronic Paramagnetic Resonance (EPR). An unambiguous shift of about 5 G in the phosphorus hyperfine coupling constant was reached, allowing a specific detection of the substrate and the product at two distinct frequencies. Being a frequency-specific imaging method, the advantage of having a shifting resonance is that both the substrate and the product are detectable and distinguishable through OMRI. This shift can be used to measure an enzyme activity by EPR in vitro or to create contrast in vivo by Overhauser-enhanced magnetic resonance imaging (Figure 4). A concentration as low as 1 nM of NE was detected in mouse BALs from a lung acute inflammation model. In humans with cystic fibrosis, concentrations of NE in the epithelial lining fluid are around 2000, the lower limit of the method even for patients with mild lung disease [47, 87]. Thus, it ensures a fast and strong signal in a few seconds in vivo.

## 3. Summary and Outlook

The rapid expansion of molecular imaging technologies highlights promising prospects for early diagnosis of proteolysis. Substrate-based imaging agents have been recently shown to have strong values for NE imaging as biomarker of inflammation. Significantly, strategies using substrate coupled to an original nitroxide in OMRI exhibit multiple advantages. Totally noninvasive, OMRI values are highly resolved and highly sensitive. Recently, another type of nitroxide was used as theranostic approach for the treatment of solid tumors. This smart agent named “Alkoxynamine” can, in vitro, spontaneously undergo hemolysis producing a highly reactive alkyl agent which in turn would induce cancer cell death and a stable nitroxide which would serve as imaging contrast agent by OMRI [88]. NE activity imaging could potentially take advantage of the enhancement of other MRI methods. Chemical exchange saturation transfer (CEST) agent as well as fluorine magnetic resonance spectrometry have been designed to detect the catalytic activity of several proteases, respectively [89–94].

Application to human diagnosis will require further development in terms of specificity and localization. Unnatural amino acid-recognition sequences could overcome such concerns by enhancing the specificity of proteolysis. On the other hand, ABPs, as covalently linked to the target protease (NE), lead to a prolonged retention at the inflamed site. This property contrasts with the irremediable diffusion of substrate-based probe.

Hence, the use of NE probes may ultimately lead to an easy methodology consisting in new diagnostic tools functioning through noninvasive protocols [95] for NE activity quantification in lung disorders especially those where this protease is regarded as the primary suspect [8]. The selectivity for other overexpressed proteases during pathologies, such as the matrix metalloproteinase MMP-2 and MMP-9, the cysteine protease cathepsin B in solid tumors, or cathepsin G, and PR3 in inflammation, opens the door for a sensitive imaging method of any protease/inhibitor imbalance.

In addition to clinical diagnosis, evaluation of disease severity and follow-up, NE molecular imaging would also certainly have a huge potential in drug development improvement [96]. Molecular imaging techniques can be used to assess drug efficacy in a more objective way than clinical outcomes. A better control of NE activity using safe and efficient inhibitors might help to downregulate proteolytic destruction and slow disease progression. AZD9668 has already shown an attractive potential in phase II study as reversible inhibitor of human NE [97]. Very recently, BAY 85-8501 was revealed as another reversible human NE inhibitor in phase II study [98].

Although overall the molecular imaging is still at stage of development, it is expected that more advancements will be achieved in the area of molecular imaging agent, and in near future, molecular imaging techniques should drive clinical transformations. By building a complete inventory of endangered areas, proteolysis imaging would allow to respond actively in a way that leads to the optimum outcome for the patient when organs can still be preserved [99]. Molecular imaging will open a novel avenue for clinicians and simultaneously support the goal of advancing personalized medicine: “*the right prevention and treatment for the right patient at the right time*” [100].

## Figures and Tables

**Table 1:** Recapitulative set of probes for NE proteolytic activity imaging.

Probe sequence	*k*_cat_/*K*_M_(M^−1^·s^−1^)	*K*_M_(*μ*M)	*k*_cat_(s^−1^)	Nature of probe	Detection modality	Probe name	References
^99m^TC-NX21909	2 × 10^5^ (*k*_inact_)			Activity-based probe	Positron emission tomography	NX21909	[33]
^99m^TC-MAG_3_-EPI-HNE-2	2 × 10^−6^ (*K*_*i*_)			Activity-based probe	Positron emission tomography	EPI-HNE-2	[34]
Biotin-PEG(4)-Nle(*O*-bzl)-Met(O_2_)-Oic-AbuPO(Oph)_2_	1.4 × 10^7^ (*k*_obs_/*I*)	n.d	n.d	Activity-based probe	Optic (fluorogenic)	Elastase-PK101	[35, 36]
Ac-AAPV-AMC	5.8 × 10^3^	n.d	n.d	Substrate-based probe	Optic (fluorogenic)	—	[37]
MeO-Suc-AAPV-AMC	11 × 10^3^	290	3.3	Substrate-based probe	Optic (fluorogenic)	—	[37]
CFP-TSGGSGGTRQFIRWGGGGSGGTTG-YFP-HHHHHH	390 × 10^5^ (*k*_obs_/*K*_M_)	0.7 ± 0.2	27 ± 5.4 (*k*_obs_)	Substrate-based probe	Optic (fluorogenic)	Protein Biosensor IV	[38]
Abz-QPMAVVQSVPQ-EDDnp	10.9 × 10^5^	n.d	n.d	Substrate-based probe	Optic (fluorogenic)	NEmo-1 & NEmo-2	[39, 40]
						Neutrophil Elastase 680 FAST^™^	[41–45]
CNC-(O-C(O)G-NHC(O)-Suc-APA-AMC	33.5 × 10^5^	n.d	n.d	Substrate-based probe	Optic (fluorogenic)	PepNA	[46]
MeO-Suc-AAPV-(R/S)C_12_H_23_NO_5_P^▪^				Substrate-based probe	MRI (dynamic nuclear polarization)	—	[47]
*R*-isomer	9.3 × 10^5^	15 ± 2.9	14 ± 0.9				
*S*-isomer	6.4 × 10^5^	25 ± 5.4	16 ± 1.1				

n.d., no data.

## Abbreviations

ABP: Activity-based probe
AMC: 7-Amino-4-methylcoumarin
BAL: Bronchoalveolar lavage
CEST: Chemical exchange saturation transfer
CF: Cystic fibrosis
CFP: Cyan fluorescent protein
CMK: Chloromethyl ketone
CNC: Cellulose nanocrystal
COPD: Chronic obstructive pulmonary disorder
EC: Enzyme commission
EPR: Electronic paramagnetic resonance
FRET: Förster resonance energy transfer
MRI: Magnetic resonance imaging
NE: Neutrophil elastase
NET: Neutrophil extracellular trap
OMRI: Overhauser-enhanced magnetic resonance imaging
PepNA: Peptide-nanocellulose aerogel
pNA: *para*-Nitro-anilide
PR3: Proteinase 3
Ser195: Serine 195
YFP: Yellow fluorescent protein.

## Additional Points

*Highlights*. (1) No robust method exists in clinic to image active Neutrophil elastase, well known to be a biomarker of inflammation. (2) Neutrophil elastase activity imaging can be reached in vitro and in vivo using probes based either on peptide substrates or on covalent inhibitors. (3) The various probes and instrumentations for Neutrophil elastase activity imaging have distinct advantages and could be best used complementarily. (4) Improvements of probe structures and imaging instrumentation should be applied to further improve probes biodistribution and specificity. (5) Recent advances in molecular imaging open the door for a new clinical tool to image any protease/inhibitor imbalance.
